# A Direct Comparison of Placebo and Nocebo Effects on Visuospatial Attention: An Eye-Tracking Experiment

**DOI:** 10.3389/fpsyt.2019.00446

**Published:** 2019-06-21

**Authors:** Carina Höfler, Jonas Potthoff, Anne Schienle

**Affiliations:** Department of Clinical Psychology, University of Graz, Graz, Austria

**Keywords:** placebo, nocebo, eye-tracking, visuospatial attention, sham transcranial magnetic stimulation

## Abstract

**Background:** Placebo and nocebo effects on visual attention are still poorly understood. This eye-tracking study directly compared effects of sham transcranial magnetic stimulation (sTMS) that was administered along with the verbal suggestion that the treatment would either increase (placebo) or decrease (nocebo) left-sided visual attention.

**Method:** Twenty women who had reported decreased attention (nocebo responders) and 20 women who had reported increased attention (placebo responders) following sTMS completed a visual search task with three visual load levels. The task was conducted once with and once without the placebo or the nocebo (sTMS). Left-sided fixations and reaction times for left-sided targets (in comparison with right-sided targets) were analyzed.

**Results:** Contrary to the verbal suggestion, the nocebo responders showed more left-sided fixations in the nocebo condition (compared with the control condition) and responded faster to left-sided targets in the high-load condition. The placebo had no effect on fixations and reaction times.

**Conclusion:** These results indicate a more beneficial effect of a nocebo compared with a placebo for the first time. Limits and possibilities of placebo and nocebo interventions are discussed.

## Introduction

Placebos and nocebos are physically or pharmacologically inert drugs, devices, or other types of sham interventions that are able to influence various clinical and physiological outcomes related to health (1). Whereas placebos have beneficial effects on specific conditions, nocebos are associated with the occurrence of negative symptoms, the worsening of symptoms, or the prevention of improvement. Both effects are considered to be ‘context effects’ because they are mediated by diverse mechanisms, such as learning, expectations, and social cognition (1).

It has been repeatedly shown that placebos and nocebos are able to change somatic and emotional processes. The most studied phenomena, “placebo analgesia” and “nocebo hyperalgesia,” refer to the experience of either decreased or increased levels of pain after sham treatment. Other placebo/nocebo phenomena, for example, those related to perceptual processes, have been investigated less frequently and are therefore still poorly understood. A few studies have shown that placebos and nocebos are able to alter visual attention [e.g., Refs. (2–7)]. In those studies, the placebo treatments reduced visual avoidance of negative affective stimuli (4, 5, 8) and enhanced the performance on a visual search task (3). In contrast, the nocebos reduced the performance on a visual search task (3) and increased visual cortex activation during negative affective picture processing (6). Thus, there is converging evidence indicating that nocebo- or placebo-related expectations are able to influence the processing of visual inputs.

In one nocebo study on attention, a surprising effect was observed (7). Healthy individuals received sham transcranial magnetic stimulation (sTMS) along with the verbal suggestion that the treatment would elicit temporary neglect-like attention deficits in the left visual field (transitory “pseudo-neglect”). Contrary to this suggestion, in those participants who had reported experiencing attention deficits, the nocebo actually enhanced the number of left-sided fixations and facilitated target detection. These results point to a paradoxical yet positive aspect of nocebo treatment, where the suggestion of unilateral attention deficits actually provokes unilateral attention improvements (7).

This unexpected finding raises questions relating to an analogous situation: what would be the effects of a placebo sTMS combined with the verbal suggestion of a unilateral improvement in attention? In general, placebo/nocebo mechanisms are still poorly understood and controversial topics of discussion. While some findings indicate that placebos and nocebos are “evil twins” that produce effects that are counterparts of one common phenomenon [e.g., Refs. (9, 10)], others argue that placebo/nocebo responses are distinct phenomena with distinct neurobiological representations [e.g., Refs. (11, 12)].

In order to better understand both mechanisms, comparative studies, which include both placebo and nocebo conditions, are needed. In the present study, the effects of equivalent placebo and nocebo suggestions on visual-spatial attention were directly compared with each other. The study design was based on a previous nocebo study (7), which was extended by adding a placebo group. Participants completed a visual search task after being treated with a placebo or nocebo device: this device was an sTMS system, which was administered with the verbal instruction that the stimulation would either induce temporary left-sided attention improvements (placebo) or deficits (nocebo). Differences in left-sided fixation frequency, as well as reaction times for left-sided targets (in comparison with right-sided targets) during sham treatment, were compared between the placebo and the nocebo groups. Based on previous placebo studies on general visual attention [e.g., Ref. (3)], it was expected that the placebo would enhance left-sided attention as reflected by an increase in left-sided fixations and faster reactions to left-sided targets (in comparison with right-sided targets). This placebo-related improvement should be more pronounced than the previously observed increase in left-sided attention during nocebo treatment (7).

## Method

### Sample

A total of 40 right-handed healthy university students with a mean age of 21.06 years (SD = 2.58) were included in the study sample. Exclusion criteria were the presence of mental/neurological disorders, medication intake (except contraceptives), participation in a previous study with a real TMS system and attention deficits as assessed by a clinical interview, and an attention test (d2) (13). All participants had normal or corrected-to-normal vision. They were recruited *via* announcements at the university campus and gave written informed consent. The study was conducted in accordance with the Declaration of Helsinki and approved by the ethics committee of the university.

### Design and Procedure

The subjects either participated in the placebo arm of the study (*n* = 20) or in the nocebo arm (*n* = 20). The placebo arm consisted of two counterbalanced conditions (with placebo vs. without placebo). The same was true for the nocebo arm (two counterbalanced conditions: with nocebo vs. without nocebo). The two conditions were separated by approximately 1 week. The design of the study is displayed in the **Supplementary Table S1**.

**Figure 1:**
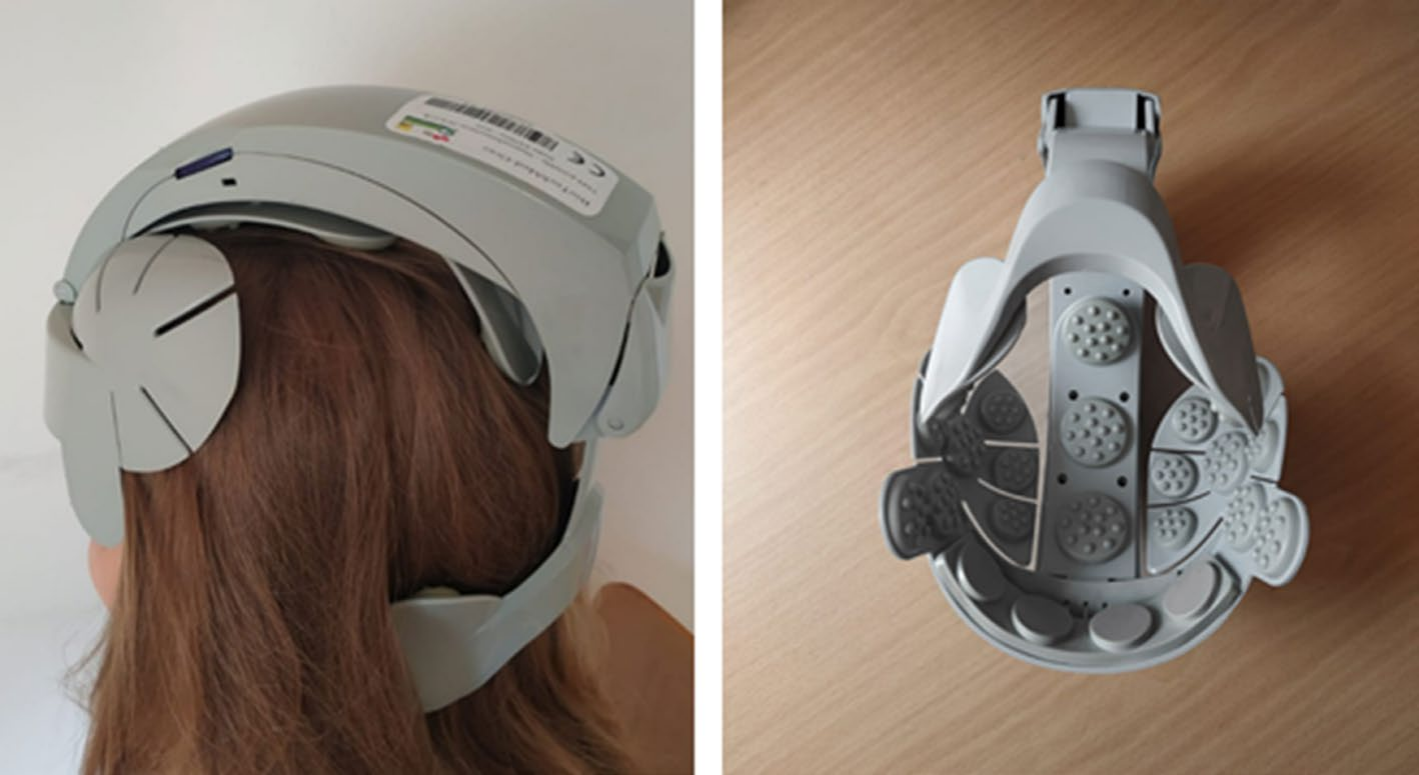
Sham device for transcranial magnetic stimulation (sTMS).

The placebo/nocebo device was an sTMS system, which was administered with the verbal suggestion that the stimulation would either induce temporary left-sided attention improvements (placebo) or deficits (nocebo). In fact, the sTMS system was a head massage tool, which induced symmetrical vibrations across the head (**Figure 1**) associated with a whirring sound. The system was presented as an innovative portable low-intensity repetitive TMS system for neurological rehabilitation. Given the increasing relevance of TMS in this field [especially in the treatment of visual neglect symptoms, e.g., Ref. (14)], this type of treatment was chosen. In order to increase the credibility of the cover story, the participants were provided with technical illustrations and a fictitious scientific article about the TMS system and its possible applications.

The sTMS system was administered for 4 min with verbal instructions either suggesting temporary left-sided attention improvements (placebo) or deficits (nocebo).

Placebo: “TMS can induce left-sided attention improvements … The visual exploration on the left side will be perceived as significantly easier and can be done faster…”

Nocebo: “TMS can induce left-sided [neglect-like] attention deficits…. The visual exploration on the left side will be perceived as significantly more challenging and exhausting…”

After the sTMS, the system was removed and the eye-tracking experiment with the visual search task started. Before and after the experiment, the affective state of the participants was assessed *via* the self-assessment manikin (1–9, 9 = happy, aroused, dominant) (15). At the end of the placebo/nocebo condition, the efficacy of the sTMS system was rated (0–100%), and the participants were asked to report experienced symptoms induced by the sTMS. At the end of the study, all participants were debriefed.

The participants of the nocebo arm were 20 “nocebo responders” [subsample of a previous study by Höfler et al. (7)], who had rated the sTMS stimulation as most effective. Effectiveness was defined as perceived change in visual attention (in the suggested direction) in percent (100% = very effective). For the placebo arm of the study, 20 women were selected from a bigger sample of 50 women (“placebo responders”). These responders did not differ from the nocebo responders in their effectiveness ratings for the sTMS (nocebo = 49.50%, SE = 3.18; placebo = 54.35%, SE = 4.03; *p* > .28). The two groups (placebo, nocebo) did not differ in mean age (nocebo = 21.00 years, SD = 2.41; placebo = 22.20 years, SD = 2.67; *p* > .14), average value of d2-attention (nocebo = 107.55, SE = 2.19; placebo = 109.65, SE = 1.48; *p* = .43), mean reaction time (nocebo = 11,209.73 ms, SE = 660.02; placebo = 10,535.56 ms, SE = 470.31; *p* = .41), and hit rate of targets in the visual search task (nocebo = 98.82%, SE = .29; placebo = 98.89%, SE = .28; *p* = .86).

We only selected “responders” for the present investigation because previous studies showed that the effects of placebos/nocebos are associated with the expected and experienced efficacy of the sham treatment [e.g., Refs. (7, 16, 17)]. Placebo/nocebo effects are mediated by diverse processes, including expectations, beliefs, and social cognition (1). In this sense, a positive/negative belief is a prerequisite for the placebo/nocebo effect to occur.

### Visual Search Task

**Figure 2:**
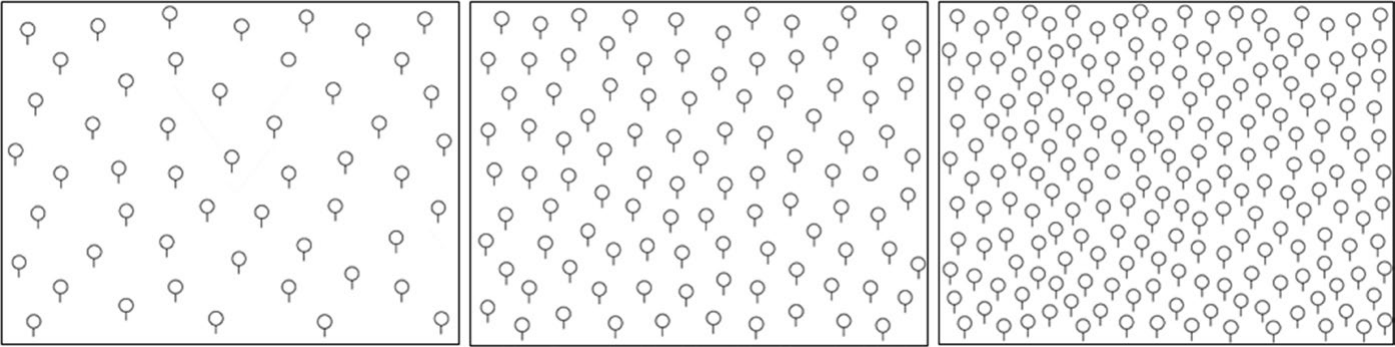
Balloon task with three visual load levels.

Participants performed a visual search task, the adapted version of the balloons test (18). The balloons task had three visual load conditions with either 50, 100, or 200 schematic black balloons depicted on a white background (**Figure 2**). Each balloon was represented by a black circle with an adjoining black vertical line originating from the bottom of the circle. The diameter of each circle was 11 mm; the line had a length of 7 mm. The balloons functioned as distractors, and one black circle without a line was the target. Participants were instructed to localize the target as fast as possible on the computer screen and confirm the detection *via* mouse click (the cursor was not visible during the search task). The mouse click was used to determine the reaction time. Subsequently, the participants were asked to point to the target to verify the correct localization. Prior to each visual load condition, a blank white screen was shown for 30 s. The sequence of the conditions was counterbalanced. Each condition comprised 12 trials; each trial had a maximum duration of 90 s. In each trial, the target had a different position oriented on a balanced 4:3 grid (six targets at each side per condition). The sequence of target location was randomized. Prior to the task, the participants performed two example tasks (target on the left/right) to get familiar with the procedure.

During the search task, two-dimensional eye movements were recorded with an SMI RED250mobile (sampling rate: 250 Hz, nine-point calibration). We calibrated both eyes and analyzed data from the eye, which produced a better spatial resolution (>0.35° visual angle). The data were only analyzed if the spatial resolution was above 0.5°. The experiment was controlled with the SMI Experiment Suite. The data were exported with SMI Begaze and customized Python scripts. For event detection, standard thresholds of the SMI BeGaze Software (Version 3.6.52) for high-speed eye-tracking data (sampling rate >200 Hz) were used: The velocity threshold for saccade detection was 40°/s. Fixations were defined by an absence of saccades and blinks (defined as moments without registered gaze positions) that lasted at least 50 ms. Participants sat about 60 cm away from the computer monitor. To minimize head movements and standardize the head position, we additionally used a chin rest. Prior to the recording, a nine-point calibration procedure was used. The paradigm was presented on a 24-in. widescreen TFT monitor with a resolution of 1,920 × 1,080 pixels.

### Data Analyses

For the analysis of the data from the balloons test, the computer screen was divided into the left and right sides (area of interest). To identify changes in directed attention due to the placebo/nocebo treatment, the percentage of left-sided (relative to right-sided) fixations was calculated (mean percent of total fixations per trial, which was within the left area of interest: values above 50% indicate a left-sided bias, values below 50% indicate a right-sided bias). Further, the lateralization index (LI) (19) of the mean reaction time for targets on the left vs. right side was determined (positive values indicate slower reactions to left-sided targets; negative values indicate faster reactions to left-sided targets).

Separate repeated-measures 3 × 2 ANOVAs were performed for the percent of the left-sided fixations and the LI of the reaction time with the within-subject factors visual load (50, 100, 200 balloons) and treatment (nocebo OR placebo, control) for the placebo and nocebo groups.

In order to compare the attention bias between the placebo and the nocebo treatments, two separate ANOVAs for the difference scores for the percent of left-sided fixations [treatment (placebo OR nocebo) minus control] and LI reaction time [treatment (placebo OR nocebo) minus control] were computed with visual load (50, 100, 200 balloons) as within-subjects factor and group (placebo, nocebo) as between-subjects factor.

To assess possible group differences in affective states, separate ANOVAs including the within-subjects factor time of measurement (before, after search task) and the between-subjects factor group were computed for the difference score of valence, arousal, and dominance [treatment (placebo OR nocebo) minus control]. We report Bonferroni adjusted *p*-values and partial eta squared (η2p) as effect size measure.

## Results

### Eye-Tracking

Descriptive statistics for the left-sided bias (fixations and reaction times) in the placebo and nocebo groups are displayed in **Table 1**.

**Table 1:** Percentages of left-sided fixations and LI reaction time (means and standard errors) in the placebo and nocebo groups (treatment minus control) for the different visual load levels.

	Placebo group						Nocebo group					
	Placebo treatment			Control			Nocebo treatment			Control		
	Low	Medium	High	Low	Medium	High	Low	Medium	High	Low	Medium	High
**Left-sided** **fixations (%)**	51.08 (1.89)	51.91 (1.81)	52.44 (2.09)	49.11 (2.07)	50.76 (2.46)	53.73 (2.08)	55.26 (2.54)	53.08 (2.70)	55.75 (2.01)	50.16 (2.81)	46.23 (2.09)	53.49 (1.89)
**Reaction time (LI)**	−.005 (.031)	.013 (.043)	−.110 (.053)	.110 (.049)	−.002 (.0538)	−.217 (.056)	−.048 (.036)	−.110 (.045)	−.246 (.035)	.013 (.053)	−.009 (.046)	−.048 (.051)

Visual load conditions (low: 50, medium: 100, high: 200 distractors); left-sided fixations (above 50% = more left-sided fixations); reaction time LI (lateralization index): negative values indicate faster reactions to left-sided targets.

*Placebo:* The conducted ANOVA for the percentages of left-sided fixations and LI reaction time in the placebo group revealed no significant main effects or interactions for the factor treatment (all *p* > .06).

*Nocebo:* In the nocebo group, the ANOVAs for fixation count [F(1, 19) = 18.65, *p* < 0.001, η2*p* = .495] and LI reaction time [F(1, 19) = 13.01, *p* = .002, η2*p* = .406] showed a significant main effect treatment. More left-sided fixations were observed, and reaction time for left-sided targets (in relation to right-sided targets) was lower in the nocebo condition compared with the control condition. The interactions treatment × visual load revealed no significant results (*p* > .18).

**Figure 3:**
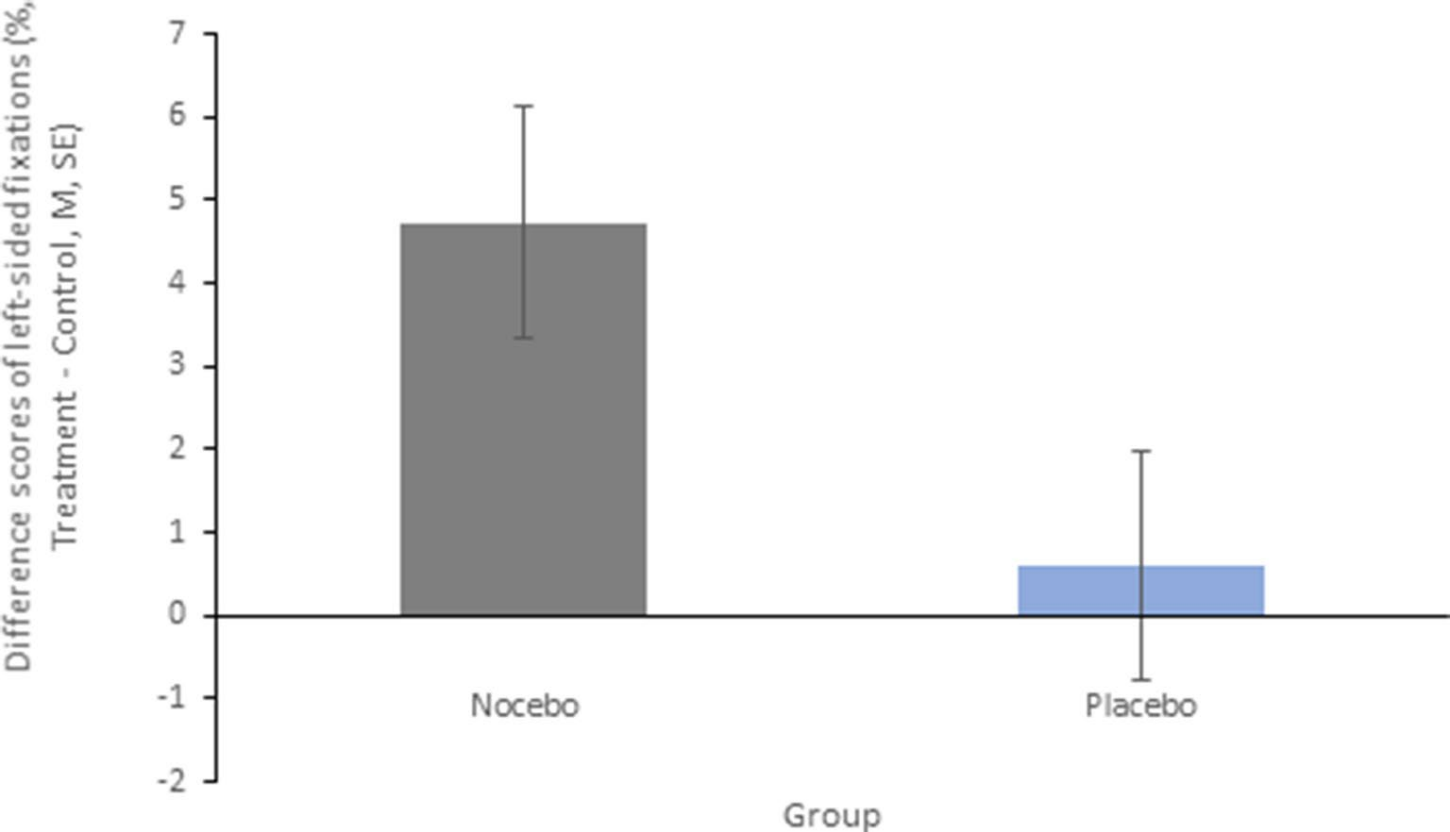
Mean difference scores and standard errors for percentages of left-sided fixations for the main effect group. Positive values indicate a higher percentage of left-sided fixations in the treatment condition (placebo or nocebo) compared with the control condition.

*Placebo vs. Nocebo:* The conducted ANOVA for left-sided fixations in the sTMS condition relative to the control condition showed a significant main effect group [F(1, 38) = 4.426, *p* = 0.042, η2*p* = .104]. The nocebo group displayed more left-sided fixations due to the treatment than the placebo group. Other effects were not significant (all *p* > .09). Means and standard errors for left-sided fixations (treatment minus control) are displayed in **Figure 3**.

**Figure 4:**
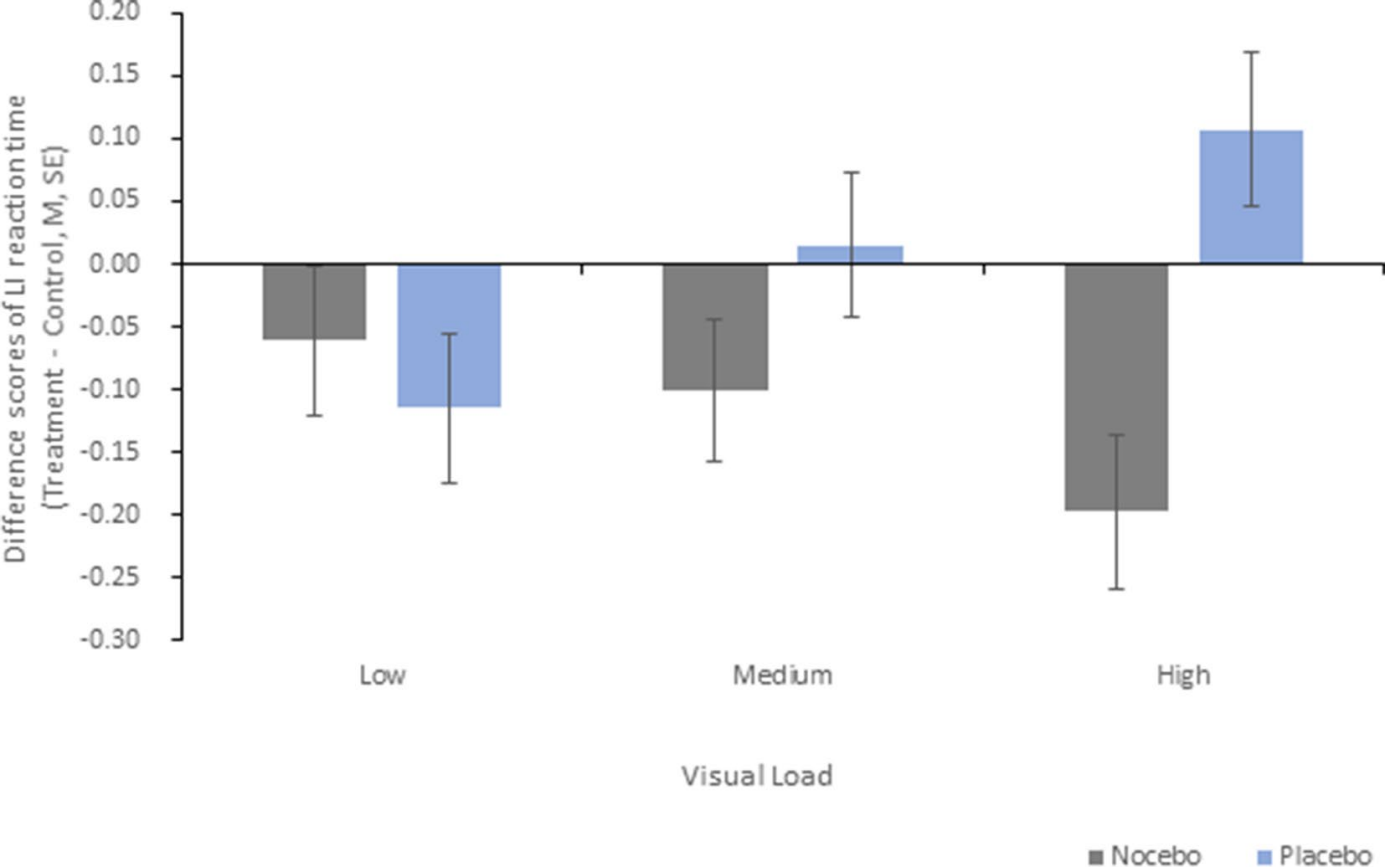
Mean difference scores and standard errors for the lateralization index reaction time for the interaction group × visual load. Negative values indicate faster reactions for left-sided targets in the treatment condition compared with the control condition.

The ANOVA for the differences in LI reaction time (treatment minus control) revealed a significant main effect group [F(1, 38) = 7.12, *p* = 0.011, η2*p* = .158] and a significant interaction group × visual load [F(2, 76) = 4.41, *p* = 0.015, η2*p* = .104]. The conducted *post hoc* *t*-tests showed that sTMS decreased response times for left-sided targets in the nocebo group in comparison with the placebo group in the high-load condition (*p* = .001) but not in the low- and medium-load conditions (both *p* > .15). The main effect visual load was not significant (*p* > .70). Means and standard errors for LI scores are shown in **Figure 4**.

### Self-Report

*Affective ratings:* The ANOVAs for the difference scores of arousal and dominance revealed a significant main effect group. The nocebo group reported higher arousal [F(1, 38) = 4.43, *p* = 0.042, η2*p* = .104] and lower dominance [F(1, 38) = 9.08, *p* = 0.005, η2*p* = .193] in the treatment relative to the control condition. The conducted ANOVA for the difference scores of valence (treatment minus control) produced no significant results (all *p* > .17). Means and standard errors for the affective ratings can be found in the **Supplementary Table S2**.

*Reported symptoms:* The following nocebo-induced symptoms were reported by the nocebo group: slower search behavior (40%), heavy eye-lid (30%), blurred vision (20%), reduced concentration (45%), and other nonspecific symptoms (60%, e.g., numbness in the left side of the body). The placebo group reported: enhanced concentration (70%), faster search behavior (45%), twitching of the eyelids (5%), perceptual changes (10%, e.g., left-sided targets appeared bigger), and other nonspecific symptoms (15%, e.g., increased sensitivity in the left side of the body) in the treatment condition.

An exploratory correlation analysis indicated that the treatment-related affective changes [treatment (placebo or nocebo) minus control] in arousal, dominance, and valence (before, as well as after the search task) were not associated with the placebo/nocebo responsiveness (percentages of left-sided fixations and LI reaction time during sTMS; all *p* > .11).

## Discussion

This eye-tracking study directly compared the effects of a placebo and a nocebo on visuospatial attention in healthy individuals. The participants reported experiencing improved attention in the placebo condition, although no changes in gaze behavior and reaction time occurred. Contrary to this, the nocebo significantly increased the number of left-sided fixations and decreased reaction time for left-sided compared with right-sided targets, especially in the condition with the highest visual load. Thus, the placebo had no effects on attention, whereas the nocebo exerted effects in the opposite direction of the verbal suggestion.

These results indicate a more beneficial effect of a nocebo, relative to a placebo, for the first time. The suggestion of a deficit in the nocebo group seemed to have prompted a need for compensation, and thus elicited a paradoxical effect. In other words, the suggestion of negative symptoms actually led to improvement. To the best of our knowledge, this is the first report on positive nocebo effects. In contrast, paradoxical placebo effects have been described before. Here, a sham treatment introduced as an agent to reduce symptoms actually made a condition worse or elicited negative side effects [for a review see (20)].

According to the present results, paradoxical interventions could be more effective than a common goal-directed placebo intervention, at least in some cases. In psychotherapy, the usefulness of paradoxical interventions has long been recognized. Particularly, when the commitment to change or therapy motivation is low, paradoxical interventions can be helpful for achieving therapy goals [e.g., Ref. (21)]. This especially applies to neuropsychological therapy where lack of compliance is a common problem in patients with disorders such as anosognosia (e.g., hemiplegia, aphasia; visual neglect). These patients are not aware of their deficit and therefore do not use, or pursue learning, compensatory strategies (22). In this specific case, nocebo interventions could open new doors in neuropsychological therapy, perhaps helping achieve positive therapy outcomes when goal-directed suggestions do not work.

The placebo group also found the treatment to be effective and experienced a subjective increase in left-sided attention. Objectively, however, this was not present. To explain this, it is very likely that the participants reduced their individual effort during the search task because of the assumed support by the sTMS treatment. This might even be considered a negative placebo effect because the participants overestimated their own attention abilities. Partly in line with this effect, when sTMS was applied, participants in the placebo group described themselves as generally more relaxed and self-confident (i.e., lower arousal and increased dominance) than those in the nocebo group. In any case, these effects portray an interesting dissociation between subjective and objective placebo/nocebo effects.

The findings of the present investigation raise basic questions regarding the possibilities and limits of placebo and nocebo treatments. It is known that placebos show differential effectiveness depending on the particular condition being treated. For example, substantial placebo effects have been found in the treatment of some disorders (e.g., depression, irritable bowel syndrome) but not in others (e.g., bacterial infections, the common cold) (23). In healthy individuals, pronounced effects have also been observed, such as when attempting to change emotional responses *via* placebo. Schienle et al. (5) administered a disgust placebo to their participants (labeled as an anti-nausea drug), while they were presented with stimuli commonly perceived as repulsive (e.g., spoiled food, excrements). The placebo reduced the intensity of experienced disgust by more than half of its original value.

In the present study, a neglect-like reaction was suggested to participants. Inducing “pseudo-neglect” (or “pseudo-unilateral attention focusing”) may be more difficult because healthy individuals have no experience with this specific phenomenon. It has been argued that direct experience (conditioning) is the most powerful way of inducing placebo-related expectancies and associated placebo responses (24); in other words, more commonly experienced reactions may be more susceptible to placebo effects. In the present investigation, a left-sided improvement/reduction of attention was suggested. This is a very specific symptom. Healthy individuals are very likely more familiar with feelings of generally reduced or increased attention and alertness. When such general changes in attention have been suggested, visual search performance was able to be altered *via* placebo/nocebo treatment (3).

It is important to acknowledge the following limitations of the present study. We only investigated women due to sex-related differences in placebo/nocebo responses [e.g., Refs. (25, 26)]. Therefore, the results cannot be generalized to men. Moreover, we did not assess or control the intake of nicotine and caffeine prior to the investigation, which might have introduced unspecific effects on general visual attention. Further, since only placebo and nocebo responders were included in the analyses, the sample size was relatively small and only allows for conclusions regarding individuals who subjectively experienced left-sided attention improvements/deficits. Finally, the nocebo group reported higher arousal and lower dominance, which may reflect a higher subjective value of the suggested left-sided deficits (as compared with left-sided improvements). However, the affective ratings were not correlated with the responsiveness to the sTMS (e.g., percentages of left-sided fixations). Therefore, it seems unlikely that the nocebo effects were mediated *via* enhanced arousal.

In summary, the present results indicate an interesting dissociation between subjectively experienced effects of placebos/nocebos and the resulting behavioral changes.

## Ethics Statement

The study was approved by the ethics committee of the University of Graz. Each participant gave written informed consent.

## Author Contributions

CH and AS designed the study and wrote the manuscript. CH and JP recruited participants for the study, collected the data, and conducted the statistical analysis of the data.

## Conflict of Interest of Statement

The authors declare that the research was conducted in the absence of any commercial or financial relationships that could be construed as a potential conflict of interest.
