# Supplementary material for: A Direct Comparison of Placebo and Nocebo Effects on Visuospatial Attention: An Eye-Tracking Experiment
Source: Front Psychiatry. 2019 Jun 21;10:446. doi: 10.3389/fpsyt.2019.00446 (PMC6597750; doi:10.3389/fpsyt.2019.00446)
Supplement: Supplementary file 1 [file DataSheet_1.docx]

**Supplementary materials**

**Table S1| Experimental design**

Foot note: Note: sTMS: sham transcranial magnetic stimulation; in each condition, the search task was performed with three different visual load levels (50, 100, 200 balloons). The sequence of both conditions and the visual load levels of the tasks were counterbalanced in each group.

|  |  | **Conditions** | |
| --- | --- | --- | --- |
| **Groups** | **Placebo Group**  **Nocebo Group** | **with sTMS** | **without sTMS** |
|  |  | Placebo | No Placebo |
|  |  | Nocebo | No Nocebo |

**Table S2| Mean affective ratings (standard errors) before and after the search task by the placebo and nocebo group in the treatment and control condition**

|  | **Placebo Group** | | | | **Nocebo Group** | | | |
| --- | --- | --- | --- | --- | --- | --- | --- | --- |
|  | **Placebo Treatment** | | **Control** | | **Nocebo Treatment** | | **Control** | |
|  | **Before** | **After** | **Before** | **After** | **Before** | **After** | **Before** | **After** |
| **Valence** | 7.05 (0.24) | 7.45 (0.22) | 7.15  (0.24) | 7.25 (0.25) | 7.60 (0.21) | 7.50 (0.25) | 7.45 (0.25) | 7.55 (0.24) |
| **Arousal** | 3.30 (0.41) | 3.10 (0.45) | 3.80  (0.36) | 2.65 (0.31) | 4.80 (0.40) | 4.25 (0.49) | 3.55 (0.32) | 3.30  (0.41) |
| **Dominance** | 5.25 (0.41) | 5.55 (0.33) | 4.80  (0.37) | 5.25 (0.35) | 5.20 (0.24) | 5.10 (0.24) | 5.55 (0.25) | 6.00 (0.31) |
